# The TBLR1/TBL1 Co‐Factor Complex Acts as a Transcriptional Checkpoint in the Brown Adipose Tissue Response to Prolonged Cold Exposure

**DOI:** 10.1096/fj.202402993RRR

**Published:** 2025-08-11

**Authors:** Sahika Cingir Köker, Foivos‐Filippos Tsokanos, Rabih El‐Merahbi, Ankush Kumar Jha, Karin Cicatelli, Peter Weber, Amit Mhamane, Doris Kaltenecker, Pauline Morigny, Anne Loft, Katarina Klepac, Adriano Maida, Claudia‐Eveline Molocea, Daniela Hass, Elena Sophie Vogl, Ana Jimena Alfaro, Wenfei Sun, Horst Zitzelsberger, Kristian Unger, Julia Szendrödi, Yongguo Li, Mauricio Berriel Diaz, Christian Wolfrum, Alexander Bartelt, Stephan Herzig, Anastasia Georgiadi

**Affiliations:** ^1^ Institute for Diabetes and Cancer (IDC), Helmholtz Center Munich (HMGU) Neuherberg Germany; ^2^ Joint Heidelberg‐IDC Transnational Diabetes Program Heidelberg University Hospital Heidelberg Germany; ^3^ Chair Molecular Metabolic Control, Medical Faculty Technical University Munich (TUM) Munich Germany; ^4^ German Center for Diabetes Research (DZD) Neuherberg Germany; ^5^ Research Unit Translational Metabolic Oncology IDC, HMGU Neuherberg Germany; ^6^ Institute of Food, Nutrition and Health (IFNH), Eidgenössische Technische Hochschule Zürich (ETH) Zurich Switzerland; ^7^ Department of Radiation Oncology University Hospital, Ludwig Maximilian University (LMU) Munich Germany; ^8^ Bavarian Cancer Research Center (BZKF) Munich Germany; ^9^ German Cancer Consortium (DKTK), Partner Site Munich Germany; ^10^ Comprehensive Cancer Center (CCC) Munich Germany; ^11^ Department of Endocrinology and Clinical Chemistry Heidelberg University Hospital Heidelberg Germany; ^12^ Institute of Pharmacology and Toxicology, University Hospital Bonn, University Bonn Bonn Germany; ^13^ German Center for Cardiovascular Research (DZHK) Partner Site Munich Heart Alliance Munich Germany; ^14^ Faculty of Medicine LMU Institute for Cardiovascular Prevention Munich Germany; ^15^ Research Department of Molecular Life Sciences, TUM Translational Nutritional Medicine, TUM School of Life Sciences Freising Germany; ^16^ Else Kröner Fresenius Center for Nutritional Medicine, TUM Munich Germany; ^17^ Head of Junior Group Endocrine Pharmacology, IDC, HMGU Neuherberg Germany

**Keywords:** brown adipose tissue, energy expenditure, HDAC3, PKA, PRDM16, TBLR1/TBL1, thermogenesis

## Abstract

Brown adipose tissue (BAT) is a key thermogenic organ, whose activation in response to cold environmental temperatures and β‐adrenergic stimulation requires the proper function of the NCOR/HDAC3 corepressor complex in brown adipocytes. The NCOR/HDAC3 complex is large and multi‐component, including the transducin beta‐like 1 (TBL1) and TBL1‐related 1 (TBLR1) proteins. Loss of TBL1 in the hepatocytes and TBLR1 in the white adipocytes has been shown to impair fasting‐ and β‐adrenergic‐induced lipolysis. However, their roles in BAT thermogenesis remain unknown. Here, we report that deletion of TBLR1 alone in brown adipocytes does not impair the adaptive thermogenic response to prolonged cold exposure. In contrast, simultaneous deletion of TBL1 and TBLR1 dampens β‐adrenergic‐induced lipolysis and mitochondrial respiration in cultured mouse brown adipocytes. Transgenic mice with UCP1‐Cre mediated double deletion of TBLR1 and TBL1 exhibit reduced whole‐body energy expenditure during prolonged cold exposure, lower core body temperature, increased appearance of unilocular adipocytes in BAT, and suppressed expression of metabolic and myogenic PRDM16 target genes. Also, we present some evidence that TBLR1 and TBL1 interact with HDAC3 and PRDM16 in brown adipocytes, potentially suggesting a direct involvement in the PRDM16‐controlled transcriptional program. These findings identify the TBLR1/TBL1 complex as a critical regulator of BAT adaptation to prolonged cold and systemic energy homeostasis, shedding light on the context‐dependent functions of corepressor complexes.

## Graphical abstract

Simultaneous deletion of TBL1 and TBLR1 in brown adipocytes leads to reduced expression of PRDM16 target genes, associated with impaired adaptive thermogenesis and defective BAT remodeling during prolonged cold exposure. While TBLR1 loss alone has minimal effects, the double knockout disrupts cold‐induced energy expenditure and promotes BAT whitening. Mechanistically, PRDM16 forms a complex with TBL1, TBLR1, and HDAC3, suggesting that this co‐repressor module acts as a transcriptional checkpoint essential for the thermogenic and structural adaptation of brown fat.

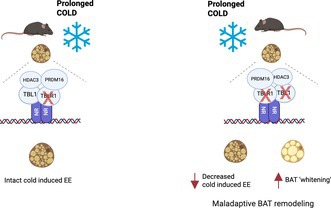

## 1 Introduction

Brown adipose tissue (BAT) is a key thermogenic organ essential for survival in small mammals exposed to low ambient temperatures. BAT thermogenesis relies on non‐shivering mechanisms [1], in which nutrients are dissipated to generate heat via uncoupling of mitochondrial electron transport from ATP synthesis, mediated by uncoupling protein 1 (UCP1) [2], as well as through UCP1‐independent futile cycling pathways [3]. Thermogenesis in BAT accounts for up to 70% of whole‐body oxidative metabolism in mice when fully recruited, making active BAT a major sink for glucose and lipoprotein‐derived lipids [4], and thereby positioning it as a major contributor to cardiometabolic health [5, 6]. Importantly, functional BAT has also been identified in adult humans [7, 8, 9], where it correlates with leanness [10] and favorable metabolic profiles [11], including lower fasting glucose [12], improved insulin‐stimulated glucose disposal [13] and reduced arterial inflammation and calcification [14]. A recent large‐scale epidemiological study further demonstrated that BAT presence was inversely associated with hyperlipidemia, type 2 diabetes, and major cardiometabolic complications [15].

Cold exposure or adrenergic stimulation activates thermogenesis in brown adipocytes in both mice and humans. High expression levels of β3‐adrenergic receptor (ADRB3) in brown adipocytes increase intracellular cAMP levels, stimulating lipolysis, UCP1 activation, and mitochondrial respiration, resulting in an acute increase in systemic oxygen consumption [16]. During prolonged cold exposure, BAT undergoes expansion through the proliferation and differentiation of adipocyte precursor cells into mature brown adipocytes [17, 18, 19], a process regulated at the transcriptional level [20]. In mice, these mature brown adipocytes primarily originate from Myf5‐positive myogenic precursors during both development and cold‐induced remodeling [21]. PRDM16, a key transcription factor, is induced by cold exposure and drives expression of thermogenic genes such as UCP1 in mature brown adipocytes, while also promoting brown adipocyte differentiation from myogenic progenitors. Loss of PRDM16 in brown adipocyte precursors impairs adipogenesis, shifts cell fate toward myogenesis, and leads to pronounced BAT whitening in vivo [22]. The transdifferentiation of myogenic precursors into brown adipocytes requires PRDM16 binding to the nuclear receptor PPARγ, a master regulator of adipogenesis. While PPARγ drives the differentiation of myogenic precursors into adipocytes, PRDM16 is required to specify the brown adipocyte lineage [23, 24].

Nuclear receptors such as PPARγ regulate gene expression by recruiting coactivator and corepressor complexes that modify chromatin accessibility [25]. One such complex—the NCOR/HDAC3 corepressor complex—has a critical role in regulating BAT thermogenesis PEVuZE5vdGU [26]. HDAC3 is essential for the acute thermogenic response in BAT [27]. TBL1 and TBLR1 are also core components of this complex in various tissues [28]. Deletion of TBL1 in hepatocytes leads to hepatic lipid accumulation [29] and TBLR1 deletion in white adipocytes impairs β‐adrenergic receptor‐mediated lipolysis and promotes lipid storage [30]. TBLR1 has been shown to physically interact with PPARγ in white adipocytes [30]. Given the central role of PPARγ in BAT function, we hypothesized that TBLR1 deletion in brown adipocytes would impair cold‐induced thermogenesis. Surprisingly, while TBLR1‐deficient mice exhibited reduced sensitivity to acute β‐adrenergic stimulation, their adaptive thermogenic response to prolonged cold exposure remained intact. In contrast, simultaneous deletion of TBL1 and TBLR1 in brown adipocytes led to impaired adaptive thermogenesis under chronic cold conditions. TBLR1 deletion showed minimal overall disruption of the BAT transcriptional program. However, double deletion of TBLR1/TBL1 dysregulated lipid metabolism gene expression, and histological analysis revealed an increase in unilocular adipocytes within BAT. Importantly, this was accompanied by reduced expression of myogenic gene markers. Moreover, TBLR1/TBL1 deletion decreased the expression of thermogenic and myogenic gene markers, all known PRDM16 targets. Also, we found that TBL1 and TBLR1 interacted with HDAC3 and PRDM16 in brown adipocytes.

Although previous studies suggest functional redundancy between TBL1 and TBLR1 [31], our findings indicate that both are required for full adaptive thermogenesis in BAT during cold exposure. Together, they coordinate PPARγ and PRDM16 transcriptional programs, which are essential for proper BAT function and systemic energy balance.

## 2 Materials and Methods

### 2.1 Animals

For studies in wild‐type mice, eight‐week‐old female C57BL6/N were obtained from Charles River Laboratories (CRL, Brussels, Belgium). Brown adipocyte‐specific TBLR1 knockout mice (BATTKO mice) were generated by crossbreeding homozygous TBLR1^fl/fl^ mice as described by Rohm et al. [30] with heterozygous UCP1‐CreERT2 mice as described by Rosenwald et al. [32]. This resulted in TBLR1^fl/fl^ UCP1‐CreERT2^+^ (BATTKO) and TBLR1^fl/fl^ UCP1‐CreERT2^−^ (Cre− control) mice, the former expressing the cre recombinase in brown adipocytes, resulting in brown adipocyte‐specific genomic recombination and knockout of TBLR1 upon tamoxifen injection. For induction of knockout, 5‐week‐old BATTKO and Cre− control mice were injected intraperitoneally (i.p.) with 100 mg/kg (approx. 2 mg/mouse/day) of tamoxifen (Sigma, Munich), dissolved in 5/6 volume sunflower oil (Sigma) and 1/6 volume ethanol, twice daily for 5 consecutive days. Mice were used at the earliest 3 weeks after the last tamoxifen injection, to minimize nonspecific side effects of tamoxifen. For the mice carrying a brown adipocyte‐specific double deletion of TBLR1 and TBL1 (BATdKO mouse model), we generated homozygous double flox mice for TBL1^fl/fl^; TBLR1^fl/fl^ and bred them with heterozygous Ucp1‐CreERT2^+^ mice. Double deletion of TBL1 and TBLR1 was performed with tamoxifen injections as described above. BATTKO and BATdKO were of a C57BL6/N genetic background. We used both male and female mouse cohorts, since both sexes showed similar phenotypes. All mice were maintained on a 12 h light/dark cycle with regular, unrestricted access to water and chow diet and at room temperature (22°C) unless stated otherwise. For cold exposure challenge experiments, mice were housed at 4°C, 5°C, 8°C, or 30°C for the periods indicated in the text. Organs and serum were collected, snap‐frozen, pulverized for further analysis, and stored at −80°C. Intra‐abdominal visceral white adipose tissue (aWAT) was dissected from the abdominal cavity, inguinal WAT (iWAT) (equivalent to subcutaneous WAT) was dissected from the layer under the skin and outside of the abdominal cavity at the hips. Brown adipose tissue (BAT) was dissected from the interscapular region. Blood serum was obtained by centrifuging blood for 30 min at 4°C and 5000 rpm. For acute CL316,243 (CL) treatment (Tocris Bioscience, Bristol), mice were injected i.p. with 1 mg/kg body weight CL in 0.9% NaCl. Indirect calorimetry was performed with mice housed individually in PhenoMaster cages (TSE Systems, Bad Homburg, Germany) at 30°C, 22°C, 4°C, or 8°C and oxygen consumption (VO_2_), carbon dioxide consumption (VCO_2_), physical activity, feeding, drinking, and body weight were recorded [33]. For the measurement of acute changes in oxygen consumption (VO_2_) in response to CL injection, mini chambers were used to minimize movement and accelerate the rate at which data points can be recorded, allowing for sufficient gas exchange rate to determine respiration in shorter time intervals than when using conventional cages. Long‐term exposure (3 days, 10 days, 3 weeks) for BATTKO was at 4°C and (3 days) for the BATdKO at 8°C. This difference in selected temperatures of the cold exposure protocol is due to the need to comply with evolving ethical standards on animal experimentation (license number ROB‐55.2‐2532.Vet_02‐21‐133). An acute cold tolerance test was done by bringing the mice from 22°C (room temperature) to 8°C for 5 h. All animal handling procedures were performed by the European Union directives and the German animal welfare act and have been approved by the institutional animal welfare officer and local authorities (governmental presidium Karlsruhe, Az. 35‐9185.81/G‐82/12 and ROB‐55.2‐2532.Vet_02‐21‐133).

Core body temperature was measured with a telemetric probe (TA‐F10, Data Sciences International, Cat# TA‐F10), which was surgically implanted into the peritoneal cavity of mice under aseptic conditions. Anesthesia was induced using 2.5% isoflurane. Before surgery, mice received a single subcutaneous dose of buprenorphine (0.1 mg/kg). Additionally, carprofen (20 mg/kg, subcutaneously [s.c.]) was administered after surgery and at least once daily for 2 days postoperatively. No surgical complications were observed, and mice were allowed a minimum recovery period of 1 week before data collection commenced. During telemetry data recording, mice remained in their home cages, which were positioned on telemetry receivers (RPC‐1, Data Sciences International). Data acquisition was carried out using an MX2 matrix (Data Sciences International), with continuous recording via PhysioTel software in PONEMAH Physiology Platform (PhysioTel system and open the software [PONEMAH Physiology Platform v.6.30]). The collected data were subsequently organized into 60‐min intervals using Microsoft Excel.

### 2.2 Primary Adipocyte Progenitor Isolation

All antibodies were obtained from eBioscience. Primary SVF‐derived Lin−Sca1+ adipocyte progenitor cells [34] were isolated from brown adipose tissue of female C57BL6/N mice at age 6–8 weeks, as performed previously in our lab [35]. Tissues were minced, digested with collagenase type II (Sigma, Munich, Germany) and magnetic sorting was used (Milteny Biotech, Bergisch Gladbach, Germany) to eliminate Lin (using anti‐CD16/32, anti‐CD31‐biotin, anti‐CD45‐biotin and anti‐Ter119‐biotin antibodies) and to enrich for Sca1.

### 2.3 Cell Culture and Differentiation

Cells were cultivated under sterile conditions at 37°C, 5% CO_2_, and 95% humidity. Media and supplements were purchased from ThermoFisher Scientific (Braunschweig, Germany) and Dulbecco's modified Eagle's medium (DMEM) with 4.5 g/L glucose was used unless stated otherwise. Brown adipocyte progenitors immortalized by SV40 T infection (preBAT cells [36]) were cultivated to confluency, and differentiation to brown adipocytes was induced by adding 5 μM dexamethasone (Dex, Sigma, Munich, Germany), 0.5 mM 3‐isobutyl‐1‐methylxanthine (IBMX, Sigma, Munich, Germany) and 0.125 mM indomethacin (Sigma, Munich, Germany) for 48 h. After that, cells were maintained in maturation medium (DMEM), 20% DMEM, 10% FBS, 1% P/S, 500 nM Dex, 172 nM insulin, and 3 nM T3 (FBS), 1% penicillin/streptomycin (P/S), 20 nM insulin (Sigma, Munich, Germany) and 1 nM 3,3′,5‐Triiodo‐L‐thyronine sodium salt (T3) (Sigma, Munich, Germany). Cells were harvested or used for assays on day 7 post‐differentiation induction. The PreBAT immortalized brown adipocyte cell line was used for experiments in passages 9 to 16. Primary adipocyte progenitors were cultivated to confluency in laminin (Santa Cruz, Heidelberg, Germany) coated cell culture plates in DMEM, 10% FBS, 1% P/S, and 10 ng/mL murine basic fibroblast growth factor (bFGF) (R&D Systems, Wiesbaden‐Nordenstadt, Germany). Differentiation was induced by exchanging the medium to DMEM, 10% FBS, 1% P/S, 500 nM Dex, 172 nM insulin, and 3 nM T3 for 48 h. After 48 h, Dex was omitted, and cells were maintained in medium DMEM, 10% FBS, 1% P/S, 172 nM insulin, and 3 nM T3. Cells were harvested or used for assays on day 8. For H‐89 (Merck, Darmstadt, Germany) treatment, cells were pre‐incubated for 1 h with/without 0 μM H‐89 and subsequently incubated for 3 h with or without 1 μM CL316,243 (Sigma‐Aldrich) and H‐89.

### 2.4 Recombinant Adenoviruses

For in vitro knockdown of TBLR1 or TBL1, adenoviruses expressing shRNAs targeting the respective mRNA or scrambled control shRNA were cloned as described previously, using the BLOCKiT Adenoviral RNAi Expression System (ThermoFischer Scientific, Braunschweig, Germany) [37, 38], produced in HEK293A cells grown in DMEM high glucose, 10% FBS, 1% (P/S), purified by ultracentrifugation in a cesium chloride (CsCl) gradient, dialyzed against 10% glycerol/PBS, and stored at −80°C.

shRNA sequences were TBLR1, 5′‐GGATGTCACGTCTCTAGATTG‐3′, TBL1, 5′‐GCGAGGATATGGAACCTTAAT‐3′, scrambled shRNA, 5′‐GATCTGATCGACACTGTAATG‐3′. Cells were transduced following public protocols [39] at a multiplicity of infection (MOI) of 10 (primary adipocytes) or 50 (preBAT cells) in the presence of 0.5 mg/mL poly‐L‐lysine (Sigma, Munich, Germany) 2 days before the induction of adipogenic differentiation.

### 2.5 Lipolysis Assay

Differentiated adipocytes were pre‐incubated for 2 h in Krebs Ringer Buffer (KRB) 115 mM NaCl, 5.9 mM KCl, 1.2 mM MgCl_2_, 1.2 mM NaH_2_PO_4_, 1.2 mM sodium sulfate (Na_2_SO_4_) (Applichem), 2.5 mM CaCl_2_, 25 mM NaHCO_3_, pH 7.4, chemicals purchased from Applichem (Darmstadt, Germany), then stimulated for 3 h in KRB containing 5 mM glucose, 5% BSA (Sigma, Munich, Germany), 25 mM HEPES (ThermoFischer Scientific, Braunschweig, Germany) and with or without 1 μM of isoproterenol (Iso, Merck, Darmstadt, Germany). Supernatants were harvested, and non‐esterified fatty acid (NEFA) levels were determined with a kit (WAKO chemicals, Neuss). Results were normalized to protein content as determined by BCA assay (ThermoFischer Scientific, Braunschweig, Germany).

### 2.6 Lipogenesis and Glucose Uptake Assay

Differentiated adipocytes were cultured in low‐glucose, serum‐free DMEM supplemented with 0.3% BSA 24 h before the assay. For the lipogenesis assay, cells were incubated for 2 h with stimulation medium KRB containing 5 mM glucose, 0.3% BSA, 20 mM HEPES, 1 μCi/mL glucose D‐[^14^C(U)] (Perkin Elmer, Rodgau, Germany) with or without 20 nM of insulin. Cells were lysed in 0.5 M sodium hydroxide (NaOH), and ^14^C incorporation into lipids was determined by triglyceride isolation from defined volumes of cell lysate, using 2:1 chloroform: methanol and subsequent scintillation counting of the lower lipid phase, determining counts per minute (cpm). Lipogenesis was calculated compared to samples without insulin treatment. Glucose uptake assays were performed, and results were calculated as described previously [35].

### 2.7 Seahorse Extracellular Flux Analysis

Using a Seahorse XF96 extracellular flow bioanalyzer (Seahorse Biosciences, Copenhagen, the Netherlands), the oxygen consumption rate (OCR) of living cells was determined in response to various stimuli. Primary BAT SVF‐derived Lin^−^Sca1^+^ adipocyte progenitor cells were isolated and plated as described above directly in laminin‐coated XF96 polystyrene (PS) microplates. Two days before the induction of adipogenic differentiation, they were transduced with adenovirus as described above and subsequently differentiated until Day 7, when the measurement was performed. PreBAT cells were transduced with adenovirus and differentiated in 15 cm dishes. On Day 7 of differentiation, adipocytes were trypsinized, counted, and seeded into XF96 cell culture plates. Cells were washed and pre‐incubated with unbuffered minimal DMEM supplemented with 2 mM L‐glutamine, 1 mM pyruvate, and 5 mM glucose (pH 7.4). For fatty acid challenge, instead of DMEM, low buffered KRB (110 mM NaCl, 4.7 mM KCl, 2 mM MgSO_4_, 1.2 mM Na_2_HPO_4_, pH 7.4) was used, supplemented with 2.5 mM glucose and 0.5 mM L‐carnitine (Sigma, Munich, Germany). Cells were stimulated with chemicals provided by the XF Cell Mito Stress Test Kit (Seahorse Bioscience, Copenhagen, the Netherlands) or 1 μM norepinephrine (NE, Sigma, Munich, Germany). For fatty acid challenge, a conjugate solution of 170 μM BSA and 1 mM sodium palmitate (Sigma, Munich, Germany) was generated by heating palmitate in a 150 mM NaCl to 70°C, adding it to 1 volume of a 37°C 340 μM BSA solution, stirring for 1 h, and adjusting the pH to 7.4. Cells were stimulated with BSA alone or with 200 μM palmitate. For primary adipocytes differentiated in XF96 microplates, data were normalized to cell density as determined by fixing cells in 95% ethanol and 5% acetic acid, staining with sulforhodamine (Sigma, Munich, Germany), elution in 10 mM Tris (pH), and determining absorption at 540 nm.

### 2.8 Quantitative RT‐PCR

Cells and tissues were harvested in QIAzol (QIAGEN, Hilden, Germany). RNA was isolated using the RNeasy Micro‐ and Mini Kit (QIAGEN, Hilden, Germany) for cells and tissues, respectively, and cDNA was synthesized with the SuperScript II Kit (ThermoFischer Scientific, Braunschweig, Germany) and Oligo (dT) primers (ThermoFischer Scientific, Braunschweig, Germany). RT‐PCR was performed using the Taqman system with Taqman Gene Expression Master Mix and specific Gene Expression Assays and an ABI StepOnePlus sequence detector (ThermoFischer Scientific, Braunschweig, Germany). Primers: Self‐made primers for *Tbl1* (*Tbl1*_for 5′ACGAGGTGAACTTTCTGGTATATCG, *Tbl1*_rev 5′GGACTGGCTAATGTGACTTTCGA, *Tbl1*_probe 5′ATCAGGTTTTTCCCACTCTGCCTTCACG), *Tblr1* (*Tblr1*_for 5′AATGGTGCCCTGGTTCCA, *Tblr1*_rev 5′AGGTGCCATCCTCATTTATGCTA, *Tblr1*_probe 5′CCGCTGCACTCATCTCTATCATCCAGAAA), *Tbp* (*Tbp*_for 5′TTGACCTAAAGACCATTTGCACTT, *Tbp*_rev 5′TTCTCATGATGACTGCAGCAAA, *Tbp*_probe 5′TGCAAGAATGCTGAATATAATCCCAAGCG), *Ucp1* (*Ucp1*_for 5′CCTTCCCGCTGGACACTG, *Ucp1*_rev 5′CCTAGGACACCTTTATACCTAATGGT, *Ucp1*_probe 5′CAAAGTCCGCCTTCAGATCCAAGGTG); *Fabp4* (mm00445880_m1).

mRNA levels relative to TATAbox binding protein (*Tbp*) expression were calculated by the delta Ct method [40].

### 2.9 RNA Sequencing

For isolation of total RNA RNeasy Micro Kit (Qiagen) was used, and samples were processed according to the manufacturer's protocol. For samples from BAT‐specific double knock‐out mice (BATdKO), library preps have been performed using Lexogen's QuantSeq 3′ mRNA‐Seq Kit FWD HT, following the manufacturer's instructions. This kit produces libraries containing a 3′ tag from each transcript and is compatible with Illumina sequencing. Finally, libraries were quality controlled on a Bioanalyzer with a Bioanalyzer High Sensitivity DNA Analysis Kit (Agilent Technologies Inc.), and quantified via fluorometric measurement (Quanti‐iT PicoGreen dsDNA Assay Kit [Invitrogen]). The libraries were pooled in equimolar proportions and sequenced as 150 bp paired‐end sequencing on an Illumina HiSeq4000 (Illumina Inc.). Raw sequencing data processing followed Lexogen's recommendations. In short, on average 4.5 M reads per sample were generated, and only the forward read was used for quantification. Raw reads were trimmed by BBDuk [41], and alignment was performed by STAR aligner (2.5.3a) to the mouse genome GRCm38. Read summing and quantification on the gene‐level was computed by Rsubread. We used the count‐based expression matrix for our analysis. The resulting count‐based expression matrix was subjected to extensive quality control based on expression distribution, correlation analysis, and PCA; outliers were then removed. Differential gene expression analysis was performed using DESeq2 standard library [42]. Over‐representation analysis was done in clusterProfiler [43]. All *p*‐values were adjusted for multiple testing using the Benjamini‐Hochberg method, with *p*_adj_ < 0.1 considered significant.

For the BAT‐specific deletion of only TBLR1 (BATTKO), BAT RNA was isolated and subjected to gene expression analysis using mouse 430.2 arrays (Affymetrix). cDNA was synthesized and hybridized according to the manufacturer's recommendations. Annotations of the arrays were performed with the CustomCDF (Version 14) with Entrez‐based gene definitions. The Raw fluorescent intensity values were normalized by quantile normalization. Differential gene expression was analyzed based on log‐linear mixed model ANOVA with a commercial software package (SAS JMP7 Genomics, version 4). Significantly differentially expressed genes were defined based on a cutoff for false discovery rate *p* < 0.05. Ingenuity software (IPA) was used for pathway analysis.

### 2.10 LISA Analysis for Transcription Factor Prediction

To identify candidate transcription factors (TFs) regulating the transcriptional changes observed in BATdKO mice exposed to 8°C, we employed the LISA (Epigenetic Landscape In Silico Subtraction Analysis) tool [44]. This approach integrates motif enrichment and ChIP‐seq binding profiles from CistromeDB, a comprehensive public database of epigenomic data, to predict TFs likely to control a given gene set.

Separate LISA analyses were performed on the lists of significantly upregulated and downregulated genes in BATdKO 8°C versus Cre− 30°C. The tool calculates TF enrichment *p*‐values based on the overlap between known TF binding sites and the regulatory regions of the input gene set, across multiple epigenomic reference datasets. The output includes multiple sample‐specific *p*‐values for each TF (derived from distinct ChIP‐seq experiments) and a combined score that ranks TFs according to the overall strength and consistency of evidence supporting their regulatory role.

### 2.11 Protein Analysis

Cells were harvested in protein extraction buffer (PEB) consisting of 25 mM Tris–HCl, 100 mM NaCl, 1 mM EDTA, 0.5% Triton‐X100, 0.5% NP‐40, 1× protease inhibitor cocktail (PIC) (Sigma, Munich, Germany), 1× phosphatase inhibitor cocktail (Sigma, Munich, Germany), 0.5 mM sodium orthovanadate (Na_3_VO_4_) (Sigma, Munich, Germany), 10 mM sodium fluoride (NaF) (Sigma, Munich, Germany) and 10 mM glycerol‐2‐phosphate (G_2_P) (Sigma, Munich, Germany) (pH 7.4). Tissue protein lysates were generated by homogenizing pre‐pulverized tissue in a tissue lyser with PEB. Twenty to fifty micrograms of protein was loaded onto 10% SDS‐polyacrylamide gels and blotted onto nitrocellulose membranes. Western blotting was performed using primary antibodies against ATGL (4126, New England Biolabs GmbH, Frankfurt, Germany), HSL (4107, New England Biolabs GmbH, Frankfurt, Germany), phosphorylated HSL (4126, New England Biolabs GmbH, Frankfurt, Germany), TBL1 (ab24548, Abcam, Cambridge, UK), TBLR1 (NB600‐270, R&D Systems GmbH, Wiesbaden Nordenstadt, Germany), VCP (ab11433, Abcam, Cambridge, UK), and UCP1 (Calbiochem, order no. 662045). Band intensity quantification was done with ImageLab software.

For co‐immunoprecipitation (co‐IP), mouse primary adipocyte progenitors from the SVF cells or PreBAT cells were differentiated to brown adipocytes and used for experiments at 8 days post‐differentiation induction. At day 8, cells were lysed in IP buffer (50 mM Tris–HCl, 1 mM EDTA, 150 mM NaCl, 1% Triton‐X100, pH 7.4). Total protein was quantified using the BCA Pierce kit (cat. Number 23225, ThermoScientific), and 500 μg of total cell lysate was brought into a volume of 400 μL of IP solution (input). The cell lysate was then mixed with 4 μg of TBL1XR1/TBLR1 or IgG isotype control antibody and rotated at 4°C overnight. The next day, 40 μL of prewashed agarose beads (ROC‐PAG50‐00‐0002) were added and rotated at 4°C for 2 h. Samples were then centrifuged at 1500× *g* for 3 min at 4°C. A portion of the supernatant (flow‐through) was collected and stored for analysis. The beads were washed four times with cold IP buffer. After the final wash, beads were resuspended in 20 μL of 3× SDS sample buffer and boiled at 95°C for 7 min. Samples were centrifuged briefly, and the supernatants (pull‐down fraction was collected and used for Western blot analysis). A 4% of the input was loaded on the gel. Materials used in the co‐IP experiment: SEPHAROSE (TM) PROTEIN A/G, Sepharose 2 mL: catalogue number, ROC‐PAG50‐00‐0002. PRDM16 Polyclonal Antibody (LIFE TECHNOLOGIES: PA520872), TBL1XR1/TBLR1 (D4J9C) Rabbit mAb: 74499S, TBL1x: Proteintech, polyclonal Rabbit: 13540‐1‐AP, HDAC3: abcam_ ab32369, Rabbit IgG control: Cell signaling, 3900, GAPDH (14C10) Rabbit mAb #2118 Cell signaling, and abcam_ab106410 against PRDM16.

### 2.12 Immunohistochemistry

Excised BAT samples were fixed in 4% (*w*/*v*) neutrally buffered formalin, embedded in paraffin, and cut into 3‐μm slices for H&E staining or immunohistochemistry. For evaluation of lipid content, H&E sections were scanned with an AxioScan.Z1 digital slide scanner (Zeiss, Jena, Germany) equipped with a 20× magnification objective. Images were evaluated using the commercially available image analysis software Definiens Developer XD 2 (Definiens AG, Munich, Germany) following a previously published procedure [45]. The calculated parameter was the ratio of the total area of lipid vacuoles per whole tissue section.

For UCP1 immunoreactivity, sections of samples were stained on a Discovery XT automated stainer (Roche Diagnostics) employing rabbit anti‐UCP1 antibody (1:1500; Abcam, ab10983). Signal detection was performed using biotinylated goat anti‐rabbit (1:750, Vector BA‐1000) as a secondary antibody and the Discovery DAB Map Kit (Ventana Medical Systems, Tucson, AZ, USA). The stained tissue sections were scanned with an AxioScan.Z1 digital slide scanner (Zeiss) equipped with a 20× magnification objective. Images were evaluated using the commercially available image analysis software Definiens Developer XD 2 (Definiens AG, Munich, Germany). The calculated parameter was the mean chromogen brown intensity of the UCP1stained tissue.

### 2.13 Statistical Analysis

Statistical analysis was performed by two‐tailed Student's *t*‐test; for RT‐PCR data, this was calculated on log10‐transformed data, and significance was assumed at *p* < 0.05. For pairwise multiple comparison procedures, an ANOVA analysis based on the Student–Newman–Keuls Method was performed.

## 3 Results

### 3.1 PKA‐Dependent Induction of TBLR1 During Cold Adaptation

Differential expression of distinct transcriptional regulators under divergent environmental conditions often reflects their causal roles in (patho) physiological adaptation. Because of the shared NE/cAMP‐driven endocrine environment, the fasting‐induced expression of TBLR1 in mouse and human WAT [30] initially prompted the hypothesis that TBLR1 may also be regulated by cold exposure. To investigate this, we exposed C57BL6/N mice to cold temperatures and assessed *Tblr1* levels in BAT, a key cold‐responsive tissue.

**FIGURE 1:**
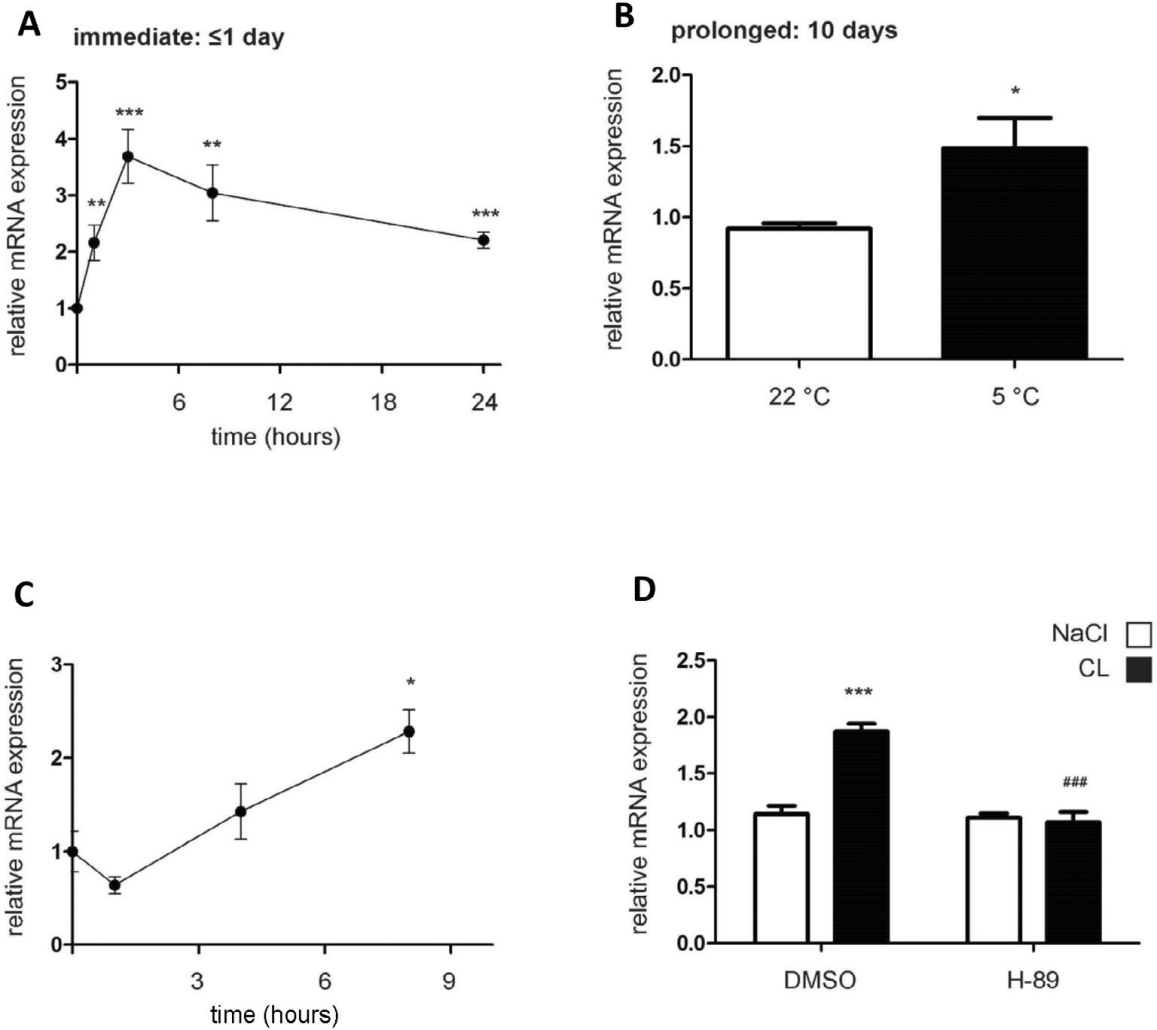
*Tblr1* mRNA expression is regulated by cold and adrenergic stimulation in BAT. (A) qPCR quantification of *Tblr1* mRNA expression in BAT of mice exposed acutely to 4°C ambient temperature, after having been acclimatized for 2 weeks at 30°C ambient temperature (*n* = 7). (B) qPCR quantified mRNA levels of *Tblr1* in BAT of mice housed at 22°C or 5°C for 10 days (*n* = 7). (C) *Tblr1* mRNA expression in BAT upon injection of 1 mg/kg body weight CL (*n* = 3). (D) qPCR quantification of *Tblr1* mRNA expression in culture‐differentiated PreBAT adipocytes upon 3 h of treatment with 1 μM CL ±50 μM H‐89, *n* = 3 independent experiments, each performed with 3–4 technical replicates per group. Mice WT, females, C57BL6/N, 8 weeks old (A–C). Relative mRNA expression was determined by qPCR using the 2^−ΔΔCT^ method. Expression was normalized to *Tbp* (reference gene), and values are shown relative to the control group. All values are expressed as means ± SEM; Statistics are Student's *t*‐test: *p < 0.05, **p < 0.01, ***p < 0.001, comparisons: (A, C) each time point versus 0 h. (D) Student's *t*‐test: ^###^ *p* < 0.001 comparison CL H‐89 versus CL DMSO and ***p < 0.001 comparison CL DMSO versus NaCl DMSO.

*Tblr1* expression peaked in BAT within the first few hours of cold exposure (Figure 1A). Notably, after the initial rise, *Tblr1* mRNA levels gradually declined over the 8‐h time course. To assess whether this induction persists under chronic cold conditions, mice were housed at 5°C for 10 days. In line with the acute response, *Tblr1* mRNA levels remained elevated in BAT after 10 days of housing at 5°C compared to animals housed at 22°C room temperature (RT) (Figure 1B).

The acute cold response is triggered by noradrenergic signaling [46], leading to increased intracellular cAMP levels [47] and activation of downstream gene expression via the PKA pathway [48]. Indeed, the intraperitoneal (i.p.) injection of the β3‐adrenergic agonist CL316,243 (CL) significantly increased *Tblr1* expression in BAT (Figure 1C). In line with these in vivo findings, the immortalized brown adipocyte cell line PreBAT cells [36] exhibited a robust upregulation of *Tblr1* after 3 h of CL treatment, which was blocked by the PKA inhibitor H‐89 (Figure 1D).

Together, these data demonstrate that cold exposure and β3‐adrenergic signaling induce the transcriptional co‐regulator TBLR1 in BAT and cultured brown adipocytes in a PKA‐dependent manner. These findings support the hypothesis that TBLR1 contributes to the coordination of thermogenesis and systemic metabolic adaptation during cold stress.

### 3.2 Single TBLR1 Loss‐of‐Function Triggers a Compensatory Phenotype Upon Long‐Term Cold Adaptation

**FIGURE 2:**
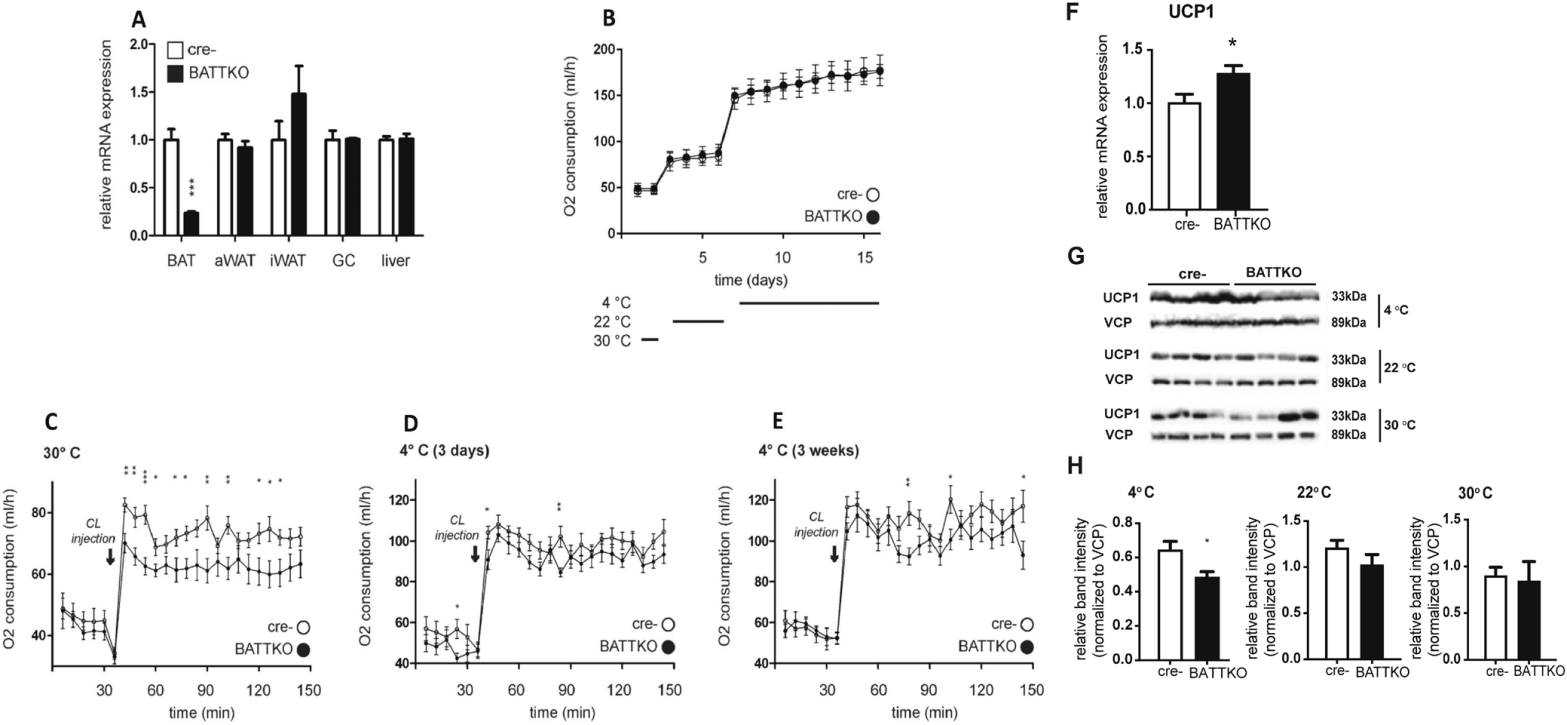
Loss of TBLR1 in brown adipocytes impairs acute adrenergic response, which is compensated during prolonged cold exposure. (A) qPCR quantification of *Tblr1* mRNA expression in brown adipose tissue (BAT), inguinal (iWAT), and abdominal (aWAT) white adipose tissue, gastrocnemius muscle (GC), and liver of BATTKO (TBLR1^fl/fl^; Ucp1‐CreERT2^+^) and control (Cre–: TBLR1^fl/fl^; Ucp1‐CreERT2^−^) mice (*n* = 4–7), 3 weeks after 5‐day tamoxifen treatment. (B) Whole‐body oxygen consumption (O_2_) in BATTKO and control mice housed individually in indirect calorimetry cages (TSE Phenomaster) and sequentially acclimated to 30°C (2 days), 22°C (4 days), and 4°C (10 days) (*n* = 6–7). (C) O_2_ consumption in mice pre‐acclimated to 30°C (3 days), followed by a single intraperitoneal injection of CL (1 mg/kg) at 30°C (*n* = 10 per group). (D, E) O_2_ consumption responses to CL injection (1 mg/kg) in mice preconditioned to cold temperature (4°C) for 3 days (D) or 3 weeks (E) (*n* = 10 per group). For (D, E) injections of CL happened after 3 days at 30°C, post cold exposure. (F) qPCR quantification of *Ucp1* mRNA expression in BAT of BATTKO and cre−mice (control) cold challenged for 10 days to 4°C, *n* = 4 mice per group. (G) Representative Western blot analysis of UCP1 protein levels in brown adipose tissue (BAT) from WT and BATTKO mice under the indicated temperature conditions. Note that samples for each temperature (4°C‐2 days, 22°C‐born and raised, 30°C‐3 days) were run on separate membranes, and thus UCP1 levels cannot be compared across temperatures. Valosin‐containing protein (VCP) was used as a loading control. (H) Quantification of UCP1 band intensity normalized to the loading control (VCP), allowing comparison between genotypes at each temperature for the WB shown (G). Relative mRNA expression was determined by qPCR using the 2^−ΔΔCT^ method. Expression was normalized to *Tbp* (reference gene), and values are shown relative to the control group. Data are presented as mean ± SEM. *p < 0.05, **p < 0.01, ***p < 0.001, BATTKO versus Cre– at matching time points.

**FIGURE 3:**
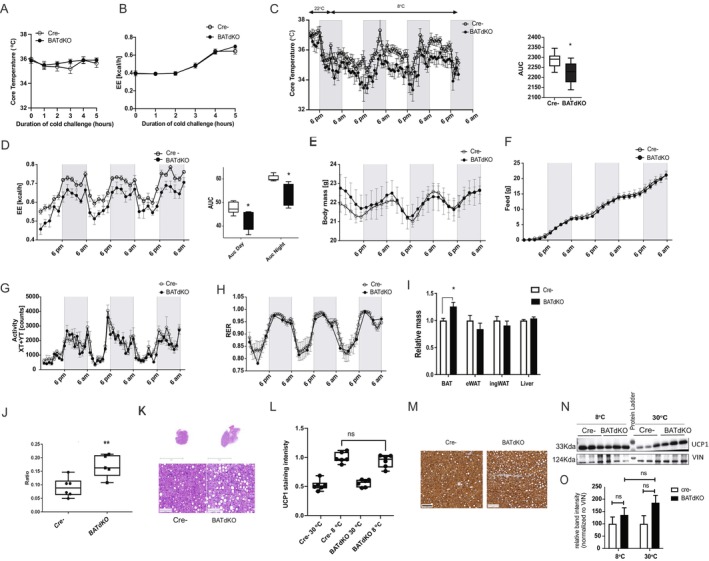
Complete loss of TBL1/TBLR1 in brown adipocytes impairs energy expenditure during prolonged cold exposure. Brown adipocyte‐specific double knockout mice (BATdKO; TBL1^fl/fl^; TBLR1^fl/fl^; Ucp1‐CreERT2^+^) and Cre−negative controls (Ucp1‐CreERT2^−^) were injected intraperitoneally with tamoxifen for 5 consecutive days and analyzed 3 weeks later. (A) Core body temperature and (B) energy expenditure (EE) (kcal/h) during acute cold challenge (8°C, for 5 h) in BATdKO and control mice. For (A, B) *n* = 6 per group. (C) Core body temperature during a 72‐h cold exposure (8°C) and area under the curve (AUC) analysis (*n* = 6 per group). (D) Energy expenditure (EE) during a 72‐h cold exposure (8°C), with representative plots and area under the curve (AUC) analysis for light and dark phases. Body mass (E), food intake (F), and physical activity (G) were recorded throughout 72 h of cold exposure. (H) Respiratory exchange ratio (RER) over the same period. (I) Relative tissue mass of BAT, epididymal WAT (eWAT), inguinal WAT (ingWAT), and liver. (J) Box plots of mean ± SEM for the ratio of total area of lipid vacuoles per whole tissue section are shown for 5–6 mice per group. (K) Representative H&E BAT images for (J). (L) Box plots of mean ± SEM for the chromogen brown intensity for UCP1, in BAT, are shown for 6 mice per group (M). Representative staining images (of L) for UCP1 in the indicated groups, in BAT. (N) Western blot against UCP1 at indicated conditions, VIN (vinculin) is used as a loading control, and relative band quantification normalized to VIN (O). (D–I) *n* = 8 male mice per group for Cre− and *n* = 10 for BATdKO. All data are presented as mean ± SEM; *p < 0.05, **p < 0.01; comparisons are between BATdKO and Cre−negative controls.

To assess the role of TBLR1 in BAT and systemic energy homeostasis, we generated thermogenic adipocyte‐specific knockout mice using a tamoxifen‐inducible Cre recombinase under the control of the UCP1 promoter [32]. Given that TBLR1 deletion in WAT is sufficient to elicit an anti‐lipolytic phenotype [30], we initially crossed Ucp1‐CreERT2^+^ mice with TBLR1^fl/fl^ animals [30]. This approach yielded brown adipocyte‐specific TBLR1 knockout mice, while WAT and other tissues remained unaffected, validating the specificity of the model (Figure 2A). Residual *Tblr1* mRNA expression in BAT is likely attributable to non‐adipocyte cell populations, as described previously [49, 50]. Throughout this study, we refer to the brown adipocyte‐specific deletion TBLR1 knockout mouse as BATTKO. Surprisingly, BATTKO mice showed no differences in whole‐body oxygen consumption at either room temperature (22°C) or cold exposure (4°C) compared to wild‐type (WT) controls (Figure 2B), suggesting that TBLR1 loss in brown adipocytes alone is insufficient to disrupt systemic energy homeostasis under chronic cold conditions. However, under thermoneutral conditions (30°C) with acute CL stimulation, BATTKO mice displayed a blunted oxygen consumption response (Figure 2C). This impaired response was no longer evident when mice were preconditioned to cold temperature for 3 days (Figure 2D) or 3 weeks (Figure 2E), indicating the activation of compensatory mechanisms that mitigate the impact of TBLR1 loss during prolonged cold adaptation, followed by an acute CL injection at 30°C.

We hypothesized that TBL1, which remains intact in BATTKO mice, may mediate this compensatory response. Supporting this hypothesis, *Tbl1* mRNA levels were also induced upon acute cold exposure (Figure S1A), and *Tbl1*, CL‐induction in brown adipocytes was blocked by PKA inhibition (Figure S1B).

At the transcriptional level, deletion of TBLR1 in BAT resulted in minimal changes in global gene expression, both at room temperature and during cold exposure (Table S1). Ucp1 mRNA levels were significantly higher in the BAT of cold‐exposed BATTKO mice compared to Cre− controls (Figure 2F). In contrast, UCP1 protein levels were modestly but significantly reduced (~20%) in BATTKO mice upon cold exposure (Figure 2G,H), while no genotype‐dependent differences were observed at 22°C or 30°C. Despite this reduction, cold‐exposed BATTKO mice showed no impairment in energy expenditure, suggesting that this level of UCP1 decrease is not sufficient to compromise thermogenic capacity, consistent with published reports [51]. Among the few significantly regulated genes in the BATTKO upon cold exposure, we noted an upregulation of several genes associated with myogenesis and muscle development, including *Atp2a1* (*Serca1*), *Myh4*, *Pygm*, *Actn3*, *Mybpc2*, *Eno3*, *Myoz1*, *Nrap1*, and *Cacna1s* (Table S1).

### 3.3 Complete Loss of the TBLR1/TBL1 Complex in BAT Affects Systemic Energy Expenditure

The absence of a cold‐induced phenotype in BATTKO mice suggested possible compensation by TBL1. To fully assess the role of TBLR1/TBL1 in BAT function, we generated double knockout mice by crossing TBLR1^fl/fl^ and TBL1^fl/fl^ animals to obtain TBLR1/TBL1 double‐floxed mice. These animals were cross‐bred with the inducible UCP1‐CreERT2^+^ animals as described in Methods to achieve brown adipocyte‐specific deletion of both genes, referred to here as BATdKO.

To examine the adaptive thermogenic response of the BATdKO animals, we performed a cold tolerance test. During acute cold exposure (8°C, for 5 h), both BATdKO and control mice (Cre−) maintained core body temperature (Figure 3A) and showed a comparable increase in energy expenditure (EE) (Figure 3B), indicating that the acute thermogenic response was preserved. However, following prolonged cold exposure at 8°C for 3 days, BATdKO mice exhibited a significant reduction in core body temperature (Figure 3C) and decreased EE across the light–dark cycle (Figure 3D) compared to controls. Body weight, food intake, physical activity, and respiratory exchange ratio remained unaltered (Figure 3E–H). These data suggest impaired BAT thermogenic activity in the BATdKO mice under prolonged cold conditions, likely due to defective BAT remodeling.

Consistent with this, BAT mass was significantly increased in BATdKO mice, while other tissues—including WAT and liver—remained unaffected (Figure 3I). This increase in BAT mass was associated with elevated lipid accumulation (Figure 3J,K), indicative of reduced lipid mobilization or utilization. Despite the thermogenic impairment, UCP1 protein levels were not significantly different between genotypes (Figure 3L–O), suggesting that thermogenesis in BATdKO mice may be impaired at the level of UCP1 activation rather than its expression or protein stability. Circulating glucose, total cholesterol, LDL cholesterol, triglycerides, and NEFA levels were also unchanged (Figure S2).

### 3.4 Loss of TBLR1/TBL1 Impairs the Metabolic Capacity of Isolated Brown Adipocytes

To determine whether deletion of the TBLR1/TBL1 complex affects brown adipocyte energetics in a cell‐autonomous manner, we employed adenoviral vectors expressing shRNAs targeting either *Tblr1*, *Tbl1*, or a non‐targeting control (AV‐shTblr1, AV‐shTbl1, or AV‐shCtrl) in isolated brown adipocytes. Experiments were conducted using both immortalized PreBAT cells [36] and primary brown adipocytes differentiated in vitro from Lin‐, Sca1+ progenitor cells [34]. Because mature adipocytes are inefficiently transduced by adenovirus, viral infection was performed 2 days prior to the induction of adipogenic differentiation. This approach yielded efficient knockdown of *Tblr1* or *Tbl1* (Figure S3A), without affecting adipogenesis, as evidenced by comparable morphology (Figure S3B) and *Fabp4* gene expression in PreBAT cells (Figure S3C).

**FIGURE 4:**
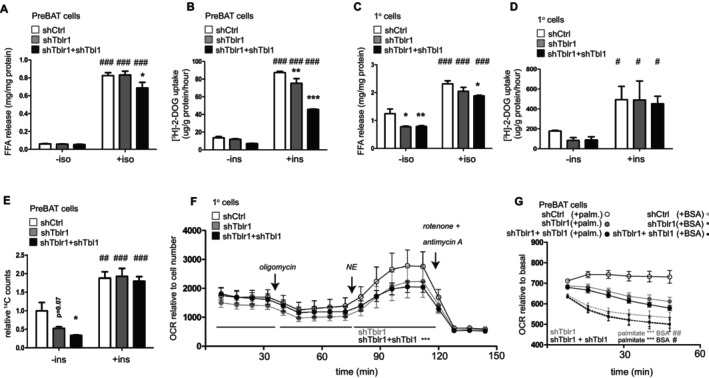
TBLR1/TBL1‐deficient brown adipocytes exhibit impaired metabolic capacity. Primary brown adipocyte progenitors (SVF‐derived) or immortalized PreBAT cells were transduced with adenoviral vectors expressing shRNA targeting *Tblr1, Tbl1*, or a control shRNA (shCtrl), and differentiated into adipocytes in vitro. (A) Free fatty acid (FFA) release from PreBAT cells 3 h after stimulation with 1 μM isoproterenol (Iso) or vehicle (−Iso), as an indicator of lipolytic activity. (B) Glucose uptake assessed by ^3^H‐2‐deoxyglucose (^3^H‐2‐DOG) incorporation into PreBAT cells under basal conditions or after stimulation with 20 nM insulin (Ins) for 20 min. FFA release (C) and ^3^H‐2‐DOG uptake (D) in primary brown adipocytes under the same treatments as in panels (A) and (B), respectively. (E) Conversion of ^14^C‐glucose into lipids in PreBAT adipocytes under basal or insulin‐stimulated conditions. (F) Mitochondrial respiration measured as oxygen consumption rate (OCR) in primary brown adipocytes stimulated with 1 μM norepinephrine (NE). Oligomycin 2 μM, rotenone, and antimycin 1 μM each. OCR was normalized to cell number and analyzed using the Seahorse extracellular flux analyzer. (G) Fatty acid oxidation in PreBAT cells measured by OCR following the addition of 200‐μM palmitate–BSA conjugate or BSA alone. OCR was normalized to basal respiration. Data are presented as mean ± SEM. *n* = 3–4 independent experiments, with 3–4 technical replicates per group. *p < 0.05, **p < 0.01, ***p < 0.001 for shRNA‐mediated knockdown versus shCtrl, within the same treatment group; ^#^ *p* < 0.05, ^##^ *p* < 0.01, ^###^ *p* < 0.001 for vehicle versus stimulation (Iso, Ins, NE, palmitate), within the same shRNA target group.

In contrast, lipolytic activity and glucose uptake were reduced in PreBAT cells lacking both TBLR1 and TBL1 compared to controls, and the impairment was more pronounced than in TBLR1 knockdown alone (Figure 4A,B). Similar reductions were observed in primary brown adipocytes (Figure 4C,D). These defects were accompanied by decreased protein levels of key lipolytic enzymes, hormone‐sensitive lipase (HSL) and adipose triglyceride lipase (ATGL), in the unstimulated state in double knockdown cells compared to shCtrl and shTblr1 conditions (Figure S3D). Furthermore, de novo lipogenesis, assessed as glucose‐to‐lipid conversion, was reduced in PreBAT cells lacking both TBLR1 and TBL1 in the unstimulated state but showed similar levels to the control upon insulin stimulation (Figure 4E). Finally, brown adipocytes deficient in TBLR1 or TBLR1/TBL1 exhibited a blunted mitochondrial response to β3‐adrenergic stimulation (Figure 4F) and to palmitate, a substrate for fatty acid oxidation (Figure 4G), as evidenced by reduced oxygen consumption. These findings suggest impaired capacity for metabolic adaptation to both adrenergic and nutrient‐derived stimuli.

Taken together, these results indicate that the TBLR1/TBL1 complex is essential for metabolic flexibility in brown adipocytes, coordinating lipolysis and glucose uptake—two processes regulated by adrenergic and insulin signaling. Reduced expression of key lipases (HSL, ATGL), along with impaired mitochondrial responses to β3‐adrenergic stimulation and fatty acid oxidation, underscores a critical role for TBLR1/TBL1 in substrate utilization and thermogenic competence. These data support the concept that TBLR1/TBL1 acts as an integrator of metabolic signaling pathways in brown adipocytes, facilitating energy balance during thermogenic activation.

However, these in vitro findings did not fully recapitulate the morphological features observed in vivo in BATdKO mice, such as increased lipid droplet size or overall lipid accumulation, suggesting additional regulatory inputs influence BAT morphology in the intact organism.

### 3.5 Complete Loss of TBLR1/TBL1 in BAT Associates With a Transcriptional Signature Indicative of Altered Lipid Handling and Impaired Function of Myogenic Progenitors

To dissect the transcriptional consequences of TBLR1/TBL1 double deletion during cold‐induced thermogenic activation, we performed RNA‐sequencing of BAT from BATdKO and control (Cre–) mice exposed to 8°C for 3 days, alongside control mice maintained at thermoneutrality (30°C). This comparison allowed us to distinguish genes altered by cold exposure from those affected by genotype.

**FIGURE 5:**
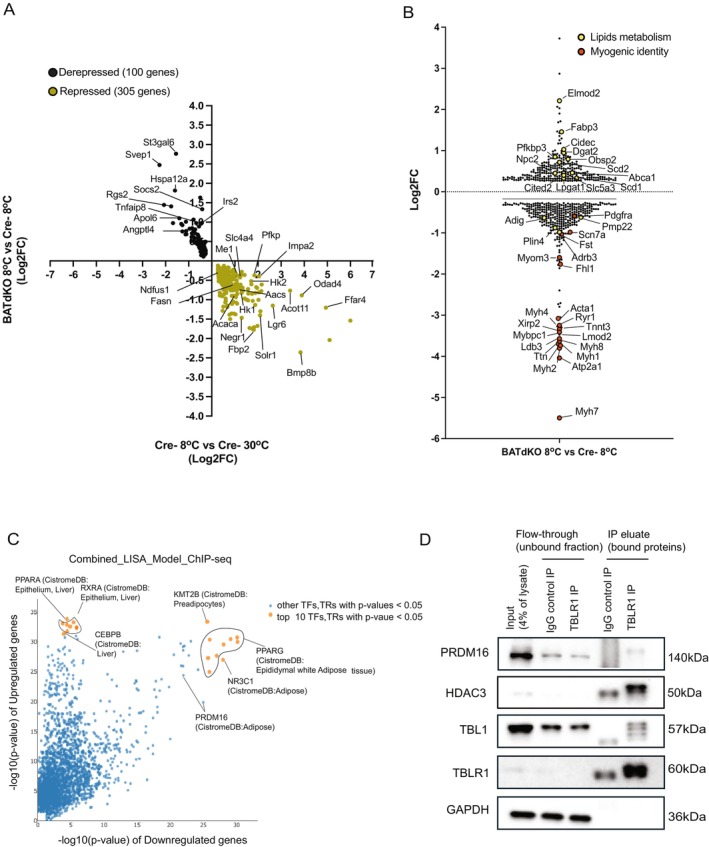
Transcriptionally regulated genes in BATdKO during cold exposure and their predicted transcriptional regulators. (A) Quadrant plot showing the log2 fold change (FC) in gene expression due to genotype (BATdKO 8°C vs. Cre− 8°C) on the *y*‐axis and cold exposure (Cre− 8°C vs. Cre− 30°C) on the *x*‐axis. Genes in the upper‐left quadrant are cold‐repressed in the Cre−, but induced in BATdKO (derepressed), while genes in the lower‐right quadrant are normally induced by cold, but suppressed in BATdKO (repressed). (B) Log2fc mRNA levels of genes altered only by the genotype upon cold exposure (BATdKO 8°C vs. Cre− 8°C). (C) Prediction hit score (−log_10_ *p*‐value) of upstream regulators, based on publicly available ChipSeq datasets, calculated by LISA.cistrome online tool. Data shown in (A, B) are normalized counts from RNAsequencing Illumina, *n* = 3–4 mice per group, males C57Bl6/N. (D) Representative WB of co‐immunoprecipitation (Co‐IP) of indicated proteins at indicated conditions. Co‐IP was performed in total cell lysates from mouse primary brown adipocytes with an antibody against TBLR1 or IgG isotype control. Antibody against PRDM16 is PRDM16 Polyclonal Antibody (LIFE TECHNOLOGIES: PA520872). Note for (D): the migration of eluted proteins may differ from input controls due to buffer composition and sample complexity, a commonly observed phenomenon in co‐immunoprecipitation assays.

Given that TBLR1 and TBL1 function as transcriptional corepressors, we hypothesized that their deletion in brown adipocytes would result in deregulated gene expression, either through derepression of normally suppressed genes or failure to induce thermogenic genes during cold exposure. We defined “derepressed” genes as those significantly upregulated in BATdKO 8°C versus Cre− 8°C (*p* < 0.05) but downregulated in Cre−8°C versus Cre− 30°C (*p* < 0.05). Conversely, “repressed” genes were significantly downregulated in BATdKO versus Cre− at 8°C, but were normally induced by cold in Cre− mice. Using this classification, we identified 100 derepressed genes and 305 repressed genes, indicating a net loss of cold‐responsive gene expression in BAT lacking TBLR1/TBL1 (Figure 5A, Extended File 1). Only a small number of genes were regulated in the same direction by both cold and genotype (Extended File 1). Additionally, we identified genotype‐specific gene changes that occurred in BATdKO at 8°C versus Cre− 8°C, but they were not significantly affected by cold in Cre− mice. These included 262 uniquely upregulated and 338 uniquely downregulated genes (Figure 5B, Extended File 2), reflecting cold‐specific responses that depended on TBLR1/TBL1 deletion per se.

Among the derepressed genes or genes which only increased in BATdKO upon cold exposure, we noted several genes involved in lipid metabolism including *Angptl4, Irs2, Dgat2, Scd1, Scd2, Npc2, Fabp3, Cidec*, and *Cited2*, and a subset associated with inflammatory signaling such as *Socs2, Apol6, Hspa12a*, *Tnfaip8*, and extracellular matrix remodeling such as *Svep1, St3gal6* (Figure 5A,B). In contrast, metabolic genes for glucose utilization such as *Hk1, Hk2*, and *Pfkp*, mitochondrial respiration and fat oxidation such as *Me1, Ndufs1*, and *Acot11*, and lipid synthesis such as *Acaca and Fasn* were repressed in BATdKO mice upon cold exposure (Figure 5A). Interestingly, we also observed strong downregulation of myogenic markers (*Acta1, Myh4, Myh7, Myh2, Myh1*) and progenitor cell markers (*Pdgfra*), suggesting impaired maintenance or differentiation of the brown adipocyte precursor pool [52] (Figure 5B). Pathway analysis revealed enrichment of lipid metabolism pathways and suppression of myogenic lineage programs (Figure S4), consistent with the established role of muscle‐like precursors in the origin of brown adipocytes—many of which are targets of PRDM16 [21, 52].

To identify upstream regulators that could explain these transcriptional shifts, we applied the LISA model using publicly available ChIP‐seq datasets, looking for predicted and ChIP‐seq validated motifs in the promoter of input genes. As input genes, we used the derepressed and upregulated genes in the cold exposed BATdKO (termed upregulated for the LISA model) and the repressed and downregulated genes in the cold exposed BATdKO (termed downregulated for the LISA model) to better distinguish transcriptional regulators that are more likely to drive the expression of upregulated or downregulated genes (Extended File 3). Among the most enriched hits, PRDM16 emerged as a top regulator of the repressed gene set (Figure 5C, Table S2 and Extended File 4). Other transcription factors (TFs) and transcriptional regulators (TRs)—including PPARγ, KMT2B, and NR3C1—were predicted to regulate both upregulated and downregulated gene sets (Figure 5C, Table S2 and Extended File 4).

PRDM16 is a well‐characterized master regulator of brown adipocyte identity, promoting thermogenesis by reprogramming myogenic precursors into brown adipocytes. It functions through interaction with various coregulators to drive the expression of thermogenic genes [23]. Notably, recent studies have shown that PRDM16 physically interacts with HDAC3 [53], modulating its activity in brown adipocytes. To test whether TBLR1/TBL1 physically interacted with HDAC3 and PRDM16 in brown adipocytes, we performed a co‐immunoprecipitation (Co‐IP) experiment using an antibody against TBLR1 to pull down the TBLR1/TBL1 complex and associated proteins. As expected, TBLR1 and TBL1 co‐precipitated, along with the co‐regulators HDAC3 and PRDM16 (Figures 5D and S5). While the IgG control lanes display bands near the molecular weights of TBLR1, TBL1, and HDAC3—likely reflecting overlap with the IgG heavy chain (~50 kDa)—the IP lanes show distinct banding patterns, and the PRDM16 band (~140 kDa) is absent from the IgG control. This supports the specificity of the interaction. While only a fraction of total PRDM16 appears to associate with the TBLR1/TBL1 complex under non‐stimulated conditions, this is consistent with the known biology of PRDM16 as a transcriptional scaffold that forms multiple, distinct complexes depending on the physiological context [54, 55, 56]. These findings may support a model in which disruption of this complex in BATdKO mice contributes to impaired cold‐induced thermogenesis.

## 4 Discussion

Transcriptional coordination is essential for the dynamic remodeling of brown adipose tissue (BAT) in response to cold exposure and for the activation of adaptive thermogenesis. In this study, we identify the transcriptional co‐factor complex TBLR1/TBL1 as a central regulator of this process. Our findings demonstrate that this complex is not only required for proper adrenergic responsiveness of mature brown adipocytes but also contributes to progenitor dynamics during prolonged cold adaptation, thereby promoting systemic metabolic adaptation to environmental cold.

Previously, we showed that mice lacking TBLR1 in white adipose tissue (WAT) exhibit impaired fasting‐induced lipolysis, partly due to reduced *Adrb3* expression and signaling, rendering them prone to high‐fat‐diet‐induced obesity [30]. Given that ADRB3 is highly expressed in BAT and is the primary mediator of adrenergic thermogenesis in mice [16], we hypothesized that loss of TBLR1 in BAT would impair thermogenesis. In support of this, TBRL1 deletion in brown adipocytes blunted oxygen consumption in response to the β3‐agonist CL‐316243—but only under thermoneutral conditions (30°C), where sympathetic nervous system (SNS) input is minimized. However, this defect was rescued after prior cold exposure, suggesting that endogenous SNS activity can compensate for the loss of TBLR1. Thus, similar to its role in WAT, TBLR1 appears necessary for intact ADRB3 signaling in BAT, though BAT possesses compensatory mechanisms that are absent in WAT.

We show that TBL1 likely contributes to these compensatory effects. While the single deletion of TBLR1 did not impair cold‐induced energy expenditure (EE), the combined deletion of TBL1 and TBLR1 significantly reduced EE and impaired cold‐induced thermogenesis, indicating a lack of functional redundancy in BAT. These findings highlight the importance of the TBLR1/TBL1 complex as a regulatory unit. Double knockout (BATdKO) mice also exhibited morphological alterations in BAT, notably increased tissue mass and accumulation of adipocytes with unilocular lipid droplets. Transcriptomic analysis revealed altered expression of genes involved in lipid droplet biology, lipid uptake, and lipid utilization, as well as lipid synthesis. However, in vitro deletion of TBLR1/TBL1 mainly supported a defect in lipid mobilization, rather than increased synthesis in brown adipocytes. Deletion of TBLR1/TBL1 in brown adipocytes reduced β‐adrenergic‐stimulated lipolysis and glucose uptake, while mitochondrial respiration remained similar between single (TBLR1) and double knockdowns. This indicates that the major effect of the double deletion lies upstream of mitochondrial activity, at the level of lipid mobilization. Consistently, HSL and ATGL protein levels were reduced only in the double knockdown, reinforcing the view that TBLR1/TBL1 acts at the level of lipolytic activation in brown adipocytes.

Interestingly, these cell‐autonomous effects did not fully replicate the morphological features observed in BAT in vivo. BATdKO mice exhibited not only larger lipid droplets but also increased numbers of unilocular adipocytes—features not reproduced in cultured cells. Brown adipocytes can arise from both adipogenic and myogenic progenitors, particularly during cold‐induced recruitment. We found broad downregulation of myogenic markers (e.g., *Myh4*, *Myh1*, *Acta1*) in BATdKO mice, suggesting altered regulation at the levels of recruitment or maintenance of this progenitor pool. These observations suggest that TBLR1/TBL1 may regulate progenitor lineage decisions in BAT. Indeed, PRDM16—a transcription factor that governs the fate switch between myogenic and brown adipogenic lineages [22] was suggested by our motif analysis to be a regulator of TBLR1/TBL1‐dependent and downregulated genes. Moreover, we confirmed that TBLR1/TBL1 physically interacts with PRDM16 and HDAC3 in brown adipocytes, supporting a model in which TBLR1/TBL1 participates in a PRDM16‐HDAC3 co‐regulatory complex, by which mechanism could control lineage‐specific and thermogenic gene expression in BAT.

However, our in vitro brown adipocyte model may lack the appropriate context to evaluate the full‐scale functional consequences of the lost interactions between TBLR1/TBL1 and the PRDM16‐HDAC3 co‐regulatory complex, as the brown preadipocyte cultures primarily consist of committed adipogenic progenitors, not bipotent myogenic ones. In vivo, Ucp1 expression can be detected early in the adipogenesis program, potentially allowing for Cre + mediated deletion of TBLR1/TBL1 in early progenitor populations, including transitioning myoblasts to brown adipocytes. Future studies using lineage tracing and myogenic progenitor‐specific deletions will be required to test whether TBLR1/TBL1 directly regulates BAT progenitor commitment or differentiation. Another interesting avenue is whether loss of TBLR1/TBL1 in mature brown adipocytes affects progenitor function indirectly via paracrine signaling.

**FIGURE 6:**
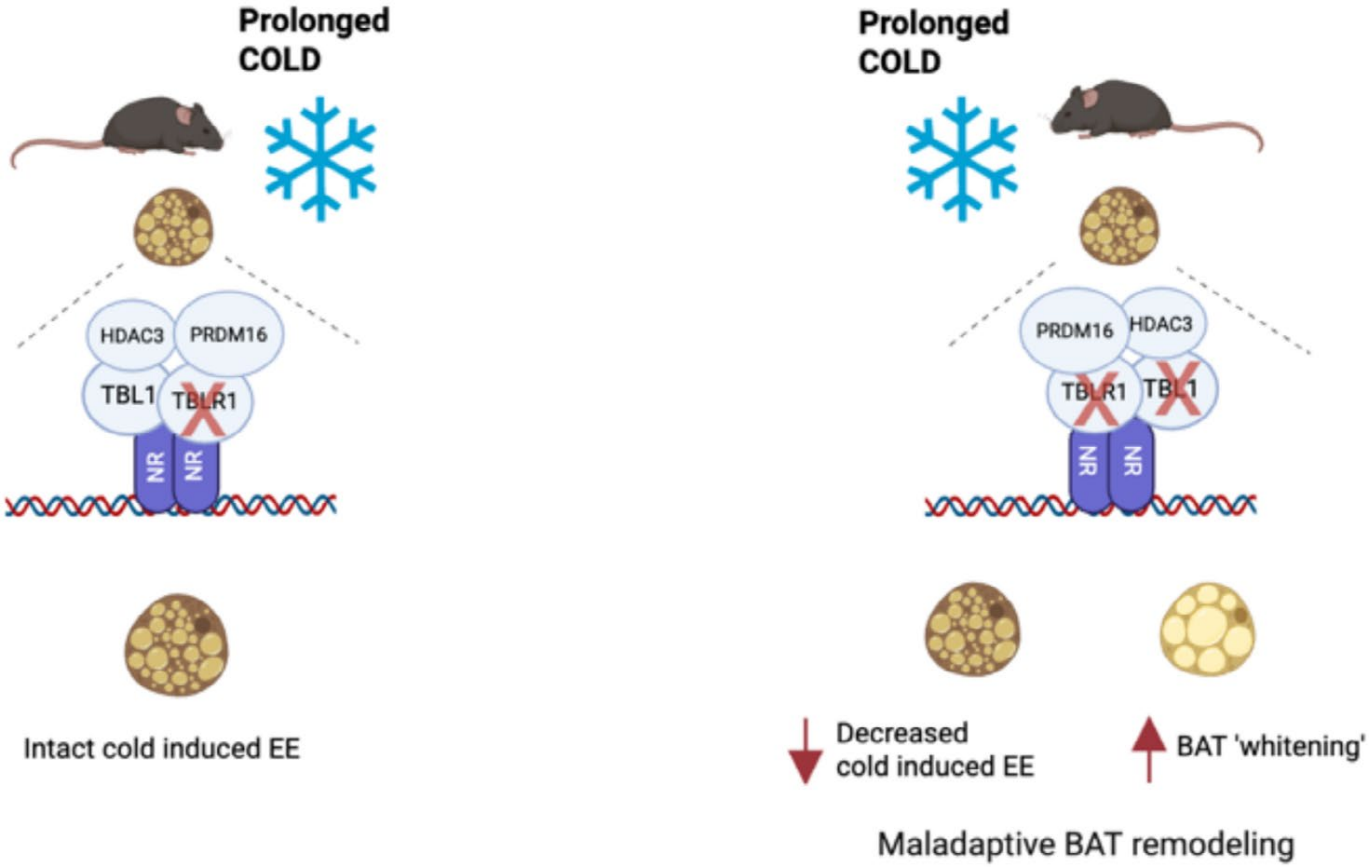
Graphical abstract of proposed model of TBLR1/TBL1 role in BAT adaptive thermogenesis upon prolonged cold exposure. Simultaneous deletion of TBLR1 and TBL1 in brown adipocytes leads to reduced expression of PRDM16 target genes, associated with impaired adaptive thermogenesis and defective BAT remodeling during prolonged cold exposure. While TBLR1 loss alone has minimal effects, the double knockout disrupts cold‐induced energy expenditure and promotes BAT whitening. Mechanistically, PRDM16 forms a complex with TBL1, TBLR1, and HDAC3, suggesting that this co‐repressor module acts as a transcriptional checkpoint essential for the thermogenic and structural adaptation of brown fat.

In summary, our study identifies the TBLR1/TBL1 transcriptional co‐factor complex as a critical regulator of cold adaptation in BAT. By integrating adrenergic signaling in mature adipocytes and controlling PRDM16‐dependent transcriptional programs, TBLR1/TBL1 emerges as a unifying checkpoint that coordinates both the acute and chronic phases of thermogenic remodeling in brown fat (Figure 6). These findings enhance our understanding of transcriptional regulation in BAT and may offer new avenues for modulating systemic energy expenditure in the context of metabolic disease.

## Author Contributions

S.C.K., F.‐F.T., R.E.‐M., A.K.J., K.C., P.W., A.Mh., D.K., P.M., A.L., K.K., A.M., C.‐E.M., D.H., E.S.V., and A.J.A. designed and performed experiments, analyzed data, and prepared the manuscript figures. W.S., H.Z., K.U., J.S., Y.L, M.B.D., C.W., and A.B. provided materials and contributed strategic input. S.H. and A.G. conceived the study, analyzed the data, and wrote the manuscript. All authors have read and approved the manuscript.

## Conflicts of Interest

The authors declare no conflicts of interest.

## Supporting information


**Data S1:** fsb270886‐sup‐0001‐DataS1.xlsx.

**Data S2:** fsb270886‐sup‐0002‐DataS2.xlsx.

**Data S3:** fsb270886‐sup‐0003‐DataS3.xlsx.

**Data S4:** fsb270886‐sup‐0004‐DataS4.xlsx.

**Figures and Tables:** fsb270886‐sup‐0005‐Supinfo.pdf.

## Data Availability

The raw data used for RNA‐seq analysis during the current study have been deposited in the NCBI Gene Expression Omnibus (GEO) under accession number GSE298802. The data will be publicly available upon publication.
