# Supplementary material for: The TBLR1/TBL1 Co‐Factor Complex Acts as a Transcriptional Checkpoint in the Brown Adipose Tissue Response to Prolonged Cold Exposure
Source: FASEB J. 2025 Aug 11;39(15):e70886. doi: 10.1096/fj.202402993RRR (PMC12337241; doi:10.1096/fj.202402993RRR)
Supplement: Supplementary file 5 — Figures and Tables: fsb270886‐sup‐0005‐Supinfo.pdf. [file FSB2-39-e70886-s003.pdf]

Supplemental information

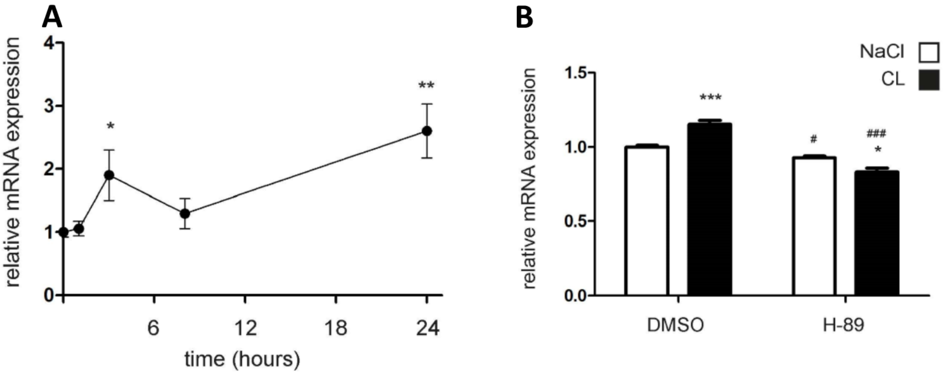

Supplementary Figure 1. (A) qPCR quantification of *Tbl1* mRNA expression levels in brown adipose tissue (BAT) of 2-week 30 °C adapted mice upon transfer to 4°C (n=7). (B) qPCR quantification of mRNA expression levels of *Tbl1* differentiated PreBAT adipocytes upon 3 hours of treatment with 1 µM CL ± 50 µM H-89, n = 3 independent experiments, each performed with three to four technical replicates per group. Relative mRNA expression was determined by qPCR using the 2^–ΔΔCT method. Expression was normalized to *Tbp* (reference gene), and values are shown relative to the control group. All values are expressed as mean ± SEM Statistics are Student's t-test. Student's t-test: *p<0.05, **p<0.01, ***p<0.001. Comparisons: (A) Each time point vs 0hr, (B) ***p<0.001 CL DMSO vs NaCl DMSO, * p<0.005 comparison CL H-89 vs NaCl H-89, ### p<0.001 comparison CL H-89 vs CL DMSO and # p<0.005 comparison NaCl H-89 vs NaCl DMSO.

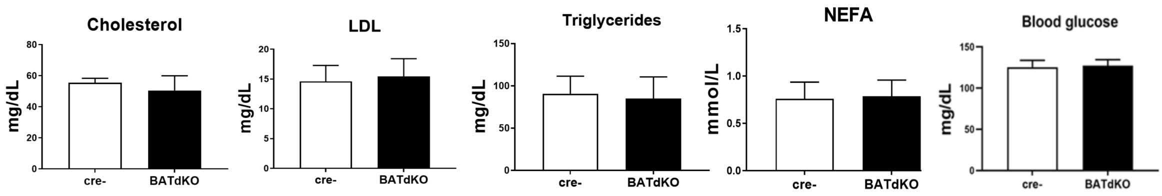

Supplementary Figure 2. Serum levels of total cholesterol, LDL cholesterol, triglycerides, non-esterified fatty acids (NEFA) and blood glucose measured at the end of the 7-day cold exposure (8ºC). All values are expressed as means ± SEM; n = 8 male mice per group for Cre- and n = 10 for BATdKO. Comparisons BATdKO vs cre- were found non-significant (p-value > 0.05), calculated with a Student's t-test, for each serum parameter.

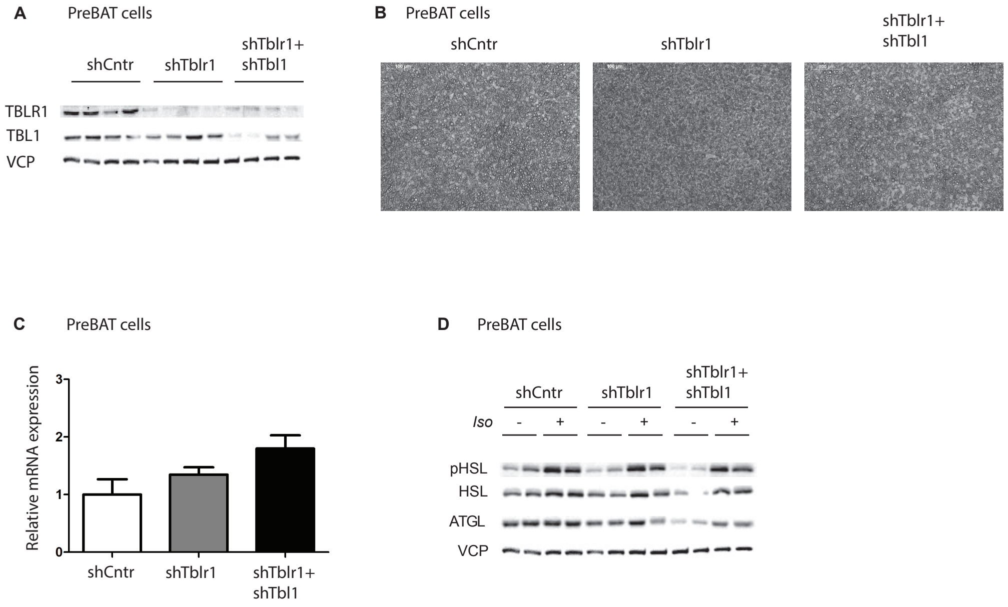

Supplementary Figure 3. preBAT cells were transduced with AV (adenovirus) expressing shTblr1 (short hairpin RNA targeting Tblr1), shTbl1 or shCtrl, and they were differentiated in vitro into adipocytes. (A) Western Blot analyses of TBLR1 and TBL1 expression, and VCP as loading control. (B) Representative images of light microscopy of day 8 of post-differentiation induction, of

preBAT adipocytes transduced with indicated AVs. (C) qPCR quantification of *Fabp4* mRNA expression levels in preBAT adipocytes transduced with the indicated AVs. Relative mRNA expression was determined by qPCR using the $2^{-\Delta\Delta CT}$ method. Expression was normalized to *Tbp* (reference gene), and values are shown relative to the control group. (D) Western Blot analyses of lipase (hormone-sensitive lipase, HSL; adipose triglyceride lipase, ATGL) expression and phosphorylation in preBAT cells upon 15 minutes treatment with 1 μM Iso or veçhile. (C) Data shown are mean ± SEM**,** n=3-4 technical replicates per experimental condition.

SUPPL. FIGURE 4

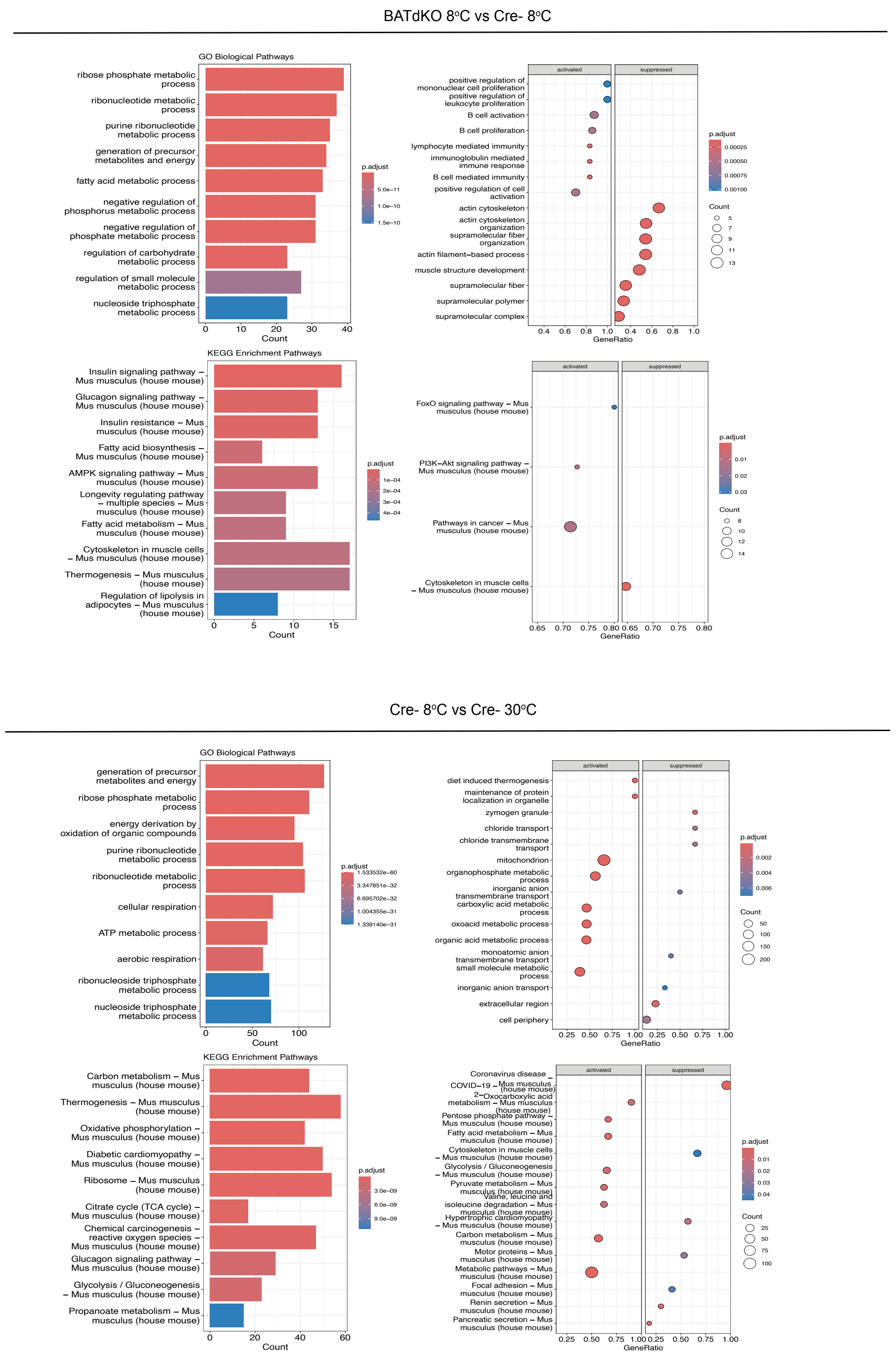

Supplementary Figure 4: Gene ontology (GO) and KEGG pathway analysis for the comparison BATdKO 8 °C vs Cre-8 °C (upper panel) and for the comparison Cre-8 °C vs Cre-30 °C (lower panel).

Bar plots show the number of genes per pathway (Counts). Dot plots show the number of genes (Counts) organized in activated and suppressed pathways.

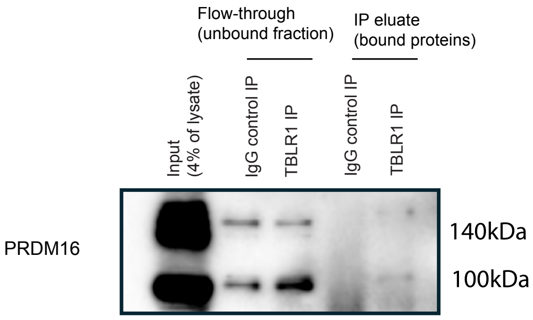

Supplementary Figure 5: Representative WB of co-immunoprecipitation (Co-IP) of PRDM16. Co-IP was performed in total cell lysates from mouse PreBAT adipocytes with an antibody against TBLR1 or IgG isotype control. Antibody against PRDM16 is ab106410 (Abcam). The two bands were detected in the input with this antibody and eluted. (Chi et al.(1) previously reported PRDM16 isoforms corresponding to sizes of 140kDa and 100kDa)

Supplementary Table 1: Fold change of significantly regulated genes between BATTKO versus WT control (Cre-), at 22 ºC and 4 ºC (for 10 days).

22ºC		
gene name	Fold change BATKO vs Cre-	p-value
Gldn	1,28	8,86E-03
Nek6	1,24	1,88E-02
Dmrt2	1,21	2,29E-02
2410066E13Rik	1,18	2,94E-05
Cdsn	1,17	4,11E-02
Apol6	1,16	2,63E-02
Ccnd1	1,16	2,68E-02
Slc6a13	1,15	3,06E-02
Col12a1	1,15	3,41E-02
Plp2	1,14	3,17E-02
H2-T23	1,14	3,76E-02
Fscn1	1,13	2,03E-02
Gp49a	1,13	3,88E-02
Il2rg	1,13	1,46E-02
Tnc	1,13	2,01E-03
Lbp	1,12	2,05E-02
Stk10	1,12	1,15E-02
Lctl	1,12	6,25E-04
1300014I06Rik	1,11	4,62E-02
Sh3tc1	1,11	3,44E-02
Hist2h3c1	0,91	2,75E-02
Slc2a12	0,91	8,96E-03
A830018L16Rik	0,89	2,36E-05
Pcdhga1	0,89	6,44E-03
Tst	0,89	2,40E-02
Apoc3	0,88	2,90E-02
Ifi27l2a	0,87	5,97E-03
Cfd	0,87	3,57E-02
Orm3	0,85	1,61E-02
Elovl3	0,83	3,39E-02

4°C		
gene name	Fold change BATKO vs Cre-	p-value
Atp2a1	1,67	9,21E-03
Myh4	1,67	3,42E-03
Pygm	1,4	8,04E-03
Actn3	1,4	2,28E-03
Mybpc2	1,25	8,21E-03
Enp3	1,23	8,16E-03
Myoz1	1,19	9,38E-03
Nrap1	1,13	8,46E-03
Cacna1s	1,11	9,73E-03
Glis3	1,1	7,69E-04
Npas2	1,1	1,25E-04
Myom1	1,1	7,40E-03
Ldlr	0,92	7,64E-03
Tubb6	0,91	6,26E-03
Hsph1	0,89	7,58E-03
Pcdhgb8	0,87	1,14E-03

Supplementary Table 2: Ranking of the top 10 candidate transcription factors (TFs) and transcription regulators (TRs) predicted to regulate genes that are upregulated and downregulated in the comparison BATdKO 8°C vs Cre- 30°C. Predictions were generated using the LISA, epigenetic modelling tool, based on transcription factor motifs and validated ChIP-seq data from CistromeDB.

	TFs and TRs	rank	1st Sample p-value	2nd Sample p-value	3rd Sample p-value	4th Sample p-value	5th Sample p-value
Upregulated genes	PPARA	1	1.49E-34	1.80E-31	6.39E-26	8.77E-19	1.13E-15
	KMT2B	2	3.91E-34	2.90E-29	6.08E-16	1.91E-15	1.97E-12
	RXRA	3	5.08E-34	7.93E-34	1.24E-33	2.70E-33	3.83E-33
	CEBPB	4	4.53E-32	1.40E-29	1.27E-27	2.67E-27	3.96E-27
	NR3C1	5	1.00E-31	3.35E-31	7.52E-31	6.22E-30	6.71E-30
	PPARG	6	1.58E-31	1.75E-31	3.42E-31	4.09E-31	9.93E-31
	NCOR1	7	5.97E-30	4.29E-29	1.50E-17	1.34E-07	1.18E-06
	THRB	8	2.76E-29	3.61E-27	1.49E-06	7.46E-06	
	CEBPA	9	1.25E-28	1.54E-28	2.38E-26	5.29E-26	1.33E-24
	NR1D1	10	3.08E-27	8.29E-27	6.28E-25	7.39E-24	9.84E-24
Downregulated genes	PPARG	1	7.11E-31	8.00E-31	8.12E-30	9.45E-29	1.08E-27
	NR3C1	2	1.17E-28	6.48E-25	8.29E-24	1.25E-22	1.27E-09
	KMT2B	3	2.81E-26	1.03E-15	4.08E-12	1.53E-11	6.49E-11
	PRDM16	4	1.05E-25	1.11E-22	5.06E-16	2.63E-04	7.37E-04
	MYOD1	5	9.71E-21	4.19E-18	1.15E-15	1.34E-15	1.13E-14
	TBX3	6	1.47E-20	1.39E-18	2.08E-03	3.80E-02	4.56E-02
	TAF7L	7	3.93E-19	1.89E-03	3.41E-02		
	EBF1	8	8.57E-19	1.07E-03	3.61E-03	3.89E-03	9.36E-03
	CEBPB	9	4.05E-18	3.44E-15	3.61E-14	1.20E-12	5.51E-12
	MYOG	10	4.17E-18	2.31E-17	5.40E-17	1.06E-11	1.18E-09

References:

1. Chi, Y. L., and Lin, J. C. (2018) RBM4a modulates the impact of PRDM16 on development of brown adipocytes through an alternative splicing mechanism. *Biochim Biophys Acta Mol Cell Res* **1865**, 1515-1525
